# Cure of Ozœna

**Published:** 1842-05

**Authors:** 


*Cure of Ozæna.*—Dr. Detmold of Hanover says, that he has never failed to cure ordinary ozæna (by which he means the chronic coryza accompanied by a stinking discharge from the nose and a flabby relaxed state of the Schneiderian membrane) by the use of an injection composed of one or two drachms of chloride of lime rubbed up in a mortar with thirteen ounces of decoction of rhatany root, and strained off after standing for half an hour. About half an ounce of this must be injected into the nose three or four times a day with a syringe whose point is sufficiently long to carry the fluid high up into the nasal passages. The use of the remedy should be accompanied by the occasional administration of purgatives. It is very beneficial also in cases of chronic otitis with offensive discharge from the ear.—*Ibid*, from *Holscher's Annalen*, B. 1804.

